# A Novel Quantitative Kinase Assay Using Bacterial Surface Display and Flow Cytometry

**DOI:** 10.1371/journal.pone.0080474

**Published:** 2013-11-15

**Authors:** Sónia Troeira Henriques, Louise Thorstholm, Yen-Hua Huang, Jennifer A. Getz, Patrick S. Daugherty, David J. Craik

**Affiliations:** 1 Institute for Molecular Bioscience, The University of Queensland, Brisbane, Queensland, Australia; 2 Department of Chemical Engineering, University of California Santa Barbara, Santa Barbara, California, United States of America; University of Torino, Italy

## Abstract

The inhibition of tyrosine kinases is a successful approach for the treatment of cancers and the discovery of kinase inhibitor drugs is the focus of numerous academic and pharmaceutical laboratories. With this goal in mind, several strategies have been developed to measure kinase activity and to screen novel tyrosine kinase inhibitors. Nevertheless, a general non-radioactive and inexpensive approach, easy to implement and adapt to a range of applications, is still missing. Herein, using Bcr-Abl tyrosine kinase, an oncogenic target and a model protein for cancer studies, we describe a novel cost-effective high-throughput screening kinase assay. In this approach, named the *BacKin* assay, substrates displayed on a *Bac*terial cell surface are incubated with *Kin*ase and their phosphorylation is examined and quantified by flow cytometry. This approach has several advantages over existing approaches, as using bacteria (i.e. *Escherichia coli*) to display peptide substrates provides a self renewing solid support that does not require laborious chemical strategies. Here we show that the *BacKin* approach can be used for kinetic and mechanistic studies, as well as a platform to characterize and identify small-molecule or peptide-based kinase inhibitors with potential applications in drug development.

## Introduction

**Figure 1:**
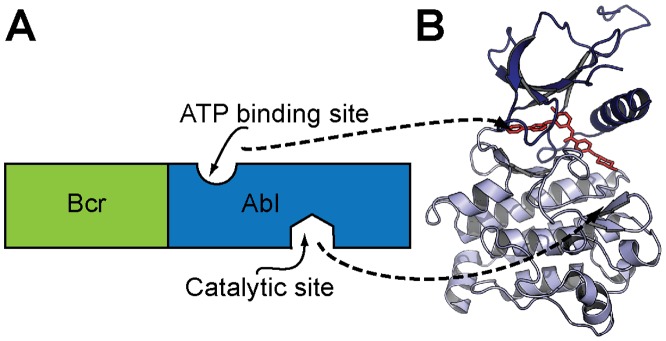
Schematic representation of the Bcr-Abl tyrosine kinase. Tyrosine kinases, such as Bcr-Abl, are phosphoryl transferases that transfer phosphate from ATP to Tyr residues on specific substrate proteins. These enzymes have an ATP-binding site that is independent of the catalytic site; when bound to ATP, they become activated and exert their activity. (A) The catalytic and ATP-binding sites of Bcr-Abl are located in the Abl domain. (B) The 3D structure of the Abl domain with imatinib (red) bound to the ATP cleft (PDB: 3k5v); imatinib occludes the ATP binding and locks the enzyme in the inactive conformation.

Tyrosine kinases (TKs) are implicated in the development of many cancers [1], [2] and are the focus of numerous drug discovery projects [3]–[5]. These critical enzymes are involved in the activation and amplification of signaling pathways within cells [2], and when deregulated can promote unregulated cell growth and cancer progression [1]. Chronic myeloid leukemia (CML) is an example in which a deregulated TK, Bcr-Abl kinase (Figure 1), gives rise to cancer [6]. As the sole molecular abnormality in the early stage of this cancer [7], Bcr-Abl kinase is an ideal oncogenic target [8] and was a landmark case in validating the clinical application of TKs as drug targets to treat cancer [9]–[11].

Imatinib was the first TK inhibitor (TKI) approved against Bcr-Abl kinase and gave rise to the so-called molecular revolution in cancer therapy [12]. This designed small molecule targets the ATP binding site in the Abl TK domain [8], [13] (Figure 1) and it is very efficacious in the treatment of CML [14]. Although remarkably effective at inhibiting cancer progression, mutations in the ATP binding site render imatinib ineffective and resistance occurs in many patients after a few years of imatinib treatment [11], [14]. As a consequence, much recent effort has been directed at identifying more potent TKIs against Bcr-Abl kinase and other oncogenic TKs [1]–[3], [5], [12], [15].

So far the discovery and development of TKIs has been based on the screening of small molecules that bind in the ATP cleft. Although a proven approach, the activity of small molecules is sensitive to kinase mutations. All human kinases have similar ATP binding pockets and therefore designing molecules targeting sites with both high specificity and a lack of susceptibility to the development of resistance is very challenging [16]. Peptide-based drugs that disrupt the interactions between TKs and their substrates [16], [17] have been proposed as alternative TKIs. The substrate-binding domain in TKs is less conserved than the ATP cleft, and peptides typically display larger contact with their targets than small molecules [2]; therefore, peptides can potentially yield higher specificity and have been a focus in our laboratory.

Assays to measure kinase activity and test and screen kinase inhibitors are essential tools for drug discovery and in the last few years several strategies have been established [18], [19]. The activity of TKs is classically measured by radiometry with radioactive phosphate transferred from [^32^P]ATP to a kinase substrate. Although the quantification of radiolabelled ATP is the ‘gold standard’ in kinase activity assays, high levels of radioactivity are required when thousands of compounds are to be screened [19]. With safety and environmental issues in mind, novel non-radioactive methods have emerged in recent years, including colorimetric methods quantifying ATP consumption [19], mass spectrometry quantification of phosphorylation by measurement of mass change upon phosphate incorporation into a peptide substrate [20], [21], an assay based on magnetic beads and matrix-assisted laser desorption/ionization [22], peptide microarray with surface plasmon resonance imaging [23] or methods using fluorescently-labeled peptides to follow fluorescence polarization, or fluorescence resonance energy transfer [24]. Although these assays are safer than those using radiolabelled ATP, they normally involve laborious chemical strategies, the use of chips, beads or expensive labeled peptides, and therefore are difficult to prepare in-house or expensive for broad application in drug discovery and lead optimization.

In the absence of an inexpensive high-throughput screening (HTS) kinase assay that could be used to identify both substrates and peptide inhibitors, measure kinase activity and compare potential lead compounds, we developed a novel kinase assay that is cost effective. In this method, substrates displayed on a bacterial cell surface are incubated with kinase and their phosphorylation is examined and quantified by flow cytometry (FC). Bcr-Abl TK, a recognized oncogenic target and a model for cancer studies, and abltide, its preferred substrate, were used to validate our method. This novel approach can be used to measure TK activity and to characterize TK inhibitors, but can also be used to identify new binders using a peptide library displayed at the bacteria cell surface and by sorting using fluorescence-activated cell sorting (FACS). It has the potential to help facilitate the rapid development of TKI drugs against Bcr-Abl kinase and can be applied for other TKs of oncogenic interest. To the best of our knowledge, this is the first kinase assay approach using peptide-displaying bacteria.

## Materials and Methods

### Peptide synthesis and purification

Based on a previously identified recognition Abl TK substrate sequence (Ile/Val-Tyr-Xaa-Xaa-Pro, where Xaa is any amino acid) [25], the peptide EAIYAAPFAKKK, named abltide, was used as an optimal substrate. Soluble abltide and the analogue [Y4F,F8Y] (EAIFAAPYAKKK), in which the Tyr4 and Phe8 residues are swapped, were synthesized using Fmoc chemistry on a Liberty Automated Microwave Peptide Synthesizer (CEM corporation, USA). The mass and the purity of the synthetic peptides were confirmed by ESI-MS and analytical reverse phase-HPLC. The peptides were quantified by absorbance at 280 nm (ε_280_  =  1490 M^−1^cm^−1^) and had a purity≥95%.

### Abltide phosphorylation followed with mass spectroscopy

The kinase activity of Bcr-Abl TK is located in the Abl domain (Figure 1). Human active Abl (121 kDa, 1067 U/mg, one unit (U) of Abl kinase defined as 1 nmol of phosphate incorporated into 50 µM abltide per minute at 30°C with a final ATP concentration of 100 µM, Merck Millipore) was used to evaluate TK activity. The activity of Abl TK was first examined using LC-MS. The time-course for phosphorylation of 60 µM of soluble abltide was followed with 0.5 U/mL Abl TK in kinase buffer (50 mM Tris-HCl, 10 mM MgCl_2_, 0.1 mM EDTA, 2 mM dithiothreitol and 0.01% (v/v) Brij 35, NEBuffer, New England BioLabs). The reaction was started by addition of ATP (500 µM final concentration) and incubated at 37°C for different times (0, 5, 10, 20, 30, 40, 50, 60, 75, 90 and 120 min) with gentle shaking. The final reaction volume was 50 µL and the reaction was stopped with 5 µL of dihydroxybenzoic acid (20 mg/mL; final concentration 2 mg/mL). Forty five µL of the reaction mixture were injected into the LC-MS. Controls without kinase or peptide were included. Separation of abltide and phosphorylated-abltide (P-abltide) was achieved using a C-18 column (Jupiter 300 5µ, 150×2.0 mm, Phenomex) using a linear AB gradient (1%/min) at 0.3 mL/min. Eluent A was 0.05% TFA (v/v) in water and eluent B was 90% acetonitrile (v/v) and 0.045% TFA (v/v) in water. With these elution conditions it was possible to separate the two peptides, and the mass was confirmed with MS. Distinction of the abltide and P-abltide in the LC-MS assay is based on the mass difference when the phosphate group is added to the Tyr residue (the molecular masses of abltide and P-abltide are 1336 and 1416 Da, respectively). The areas of the two peaks in the LC chromatogram were determined and the percentage of peptide phosphorylation calculated assuming that peak area is directly proportional to the amount of peptide in the reaction. Any contribution from the reaction mixture was discounted. Non-catalyzed phosphorylation did not occur, as confirmed in the control without kinase. The experiment was repeated independently on three different days.

### Bacterial surface display construct

**Figure 2:**
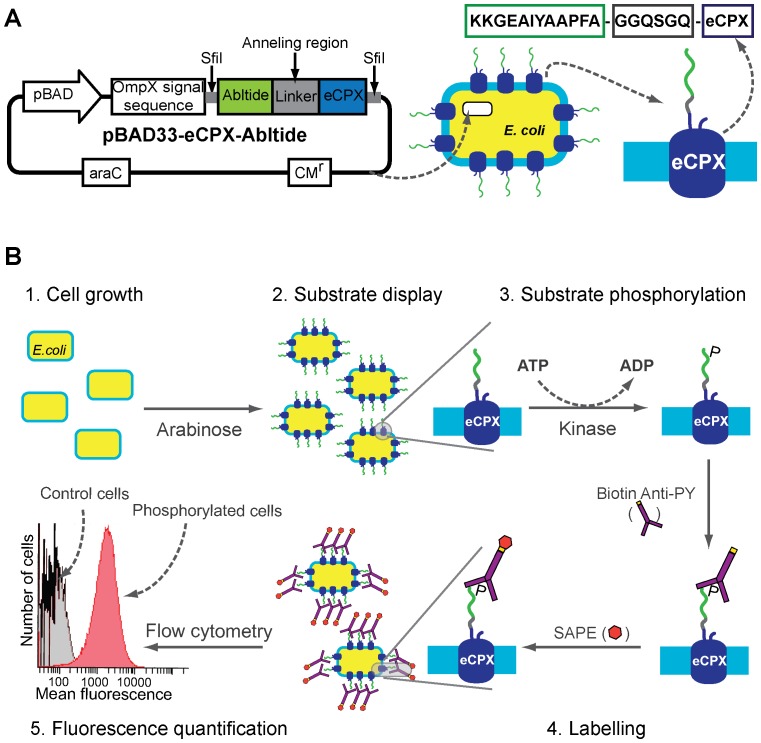
*BacKin* assay procedure. (A) Map of plasmid pBAD33-eCPX-abltide (left) and schematic representation of abltide fused to eCPX expressed on the cell surface of an *E. coli* cell transformed with pBAD33-eCPX-abltide (right). The plasmid carries the arabinose promoter pBAD and resistance to chloramphenicol (CM^r^). The recognition sites for the restriction enzyme SfiI and annealing region are show. (B) Schematic representation of the *BacKin* assay: 1. *E. coli* cells transformed with eCPX-substrate plasmid are incubated at 37°C and shaken to grow until mid log phase; 2. Substrate (e.g. abltide) expression and display on the bacteria surface is induced with arabinose; 3. Substrate phosphorylation, cells are incubated with kinase (e.g. Abl kinase) and an excess of ATP; 4. Phosphorylated substrate is labeled by incubation of cells with biotinylated-anti-phosphotyrosine antibody (biotin-anti-PY) followed by incubation with streptavidin-phycoerythrin (SAPE); 5. Mean fluorescence of kinase-treated cells is quantified by flow cytometry and compared with mean fluorescence of kinase untreated cells.

Abltide was displayed on the surface of *E. coli* by fusing the C-terminus of the sequence KKGEAIYAAPFA to the N-terminus of the enhanced circularly permuted outer protein OmpX (eCPX) display scaffold [26]. The spacer sequence GGQSGQ [26] was included to efficiently display abltide at the cell surface (Figure 2). The plasmid pBAD33-eCPX, which contains an arabinose promoter (pBAD) and resistance to chloramphenicol (Cm^r^) [26], was used to prepare the abltide construct (Figure 2A). *E. coli* strain MC1061 was transformed to display the abltide fused to eCPX. Assembly PCR was performed using KOD Hot Start DNA polymerase (Novagen) with overlapping primers including the abltide sequence, the linker, SFiI cut site and an eCPX link (GTAGCTGGCCAGTCTGGCCAGAAAAAAGGCGAAGCGATTTATGC (forward primer outside), AGGCGAAGCGATTTATGCGGCGCCGTTTGCGGGAGGGCAGTCTGGGCAGTC (forward primer inside). The primer GGCTGAAAATCTTCTCTC was used as reverse primer and pBAD33-eCPX as the template. The product of PCR reaction and the vector pBAD33-eCPX were digested with SFiI (New England Biolabs) and ligated. The ligation product (pBAD33-eCPX-abltide) was desalted and electroporated into electrocompetent *E. coli* MC1061 with 2 mm electroporation cuvette and pulse at 2.5 kV, 50 µF, and 100 Ω. Transformed cells were grown in super optimal broth supplemented with glucose and isolated clones were plated and the sequence confirmed by plasmid DNA sequencing.

### Bacterial Growth and induction conditions

An isolated clone of *E. coli* transformed with pBAD33-eCPX-abltide was grown overnight in 3 mL Luria broth supplemented with 34 µg/mL chloramphenicol (LB-CM) at 37°C. Sixty µL of overnight culture were subcultured into 3 mL of fresh LB-CM and incubated at 37°C with shaking at 225 rpm for 2 h (optical density at 600 nm is ∼0.6). To induce protein expression, 60 µL of L-arabinose 2% (w/v) (final concentration 0.04% (w/v)) and 60 µL of 100 mM EDTA (final concentration 2 mM) were added to cell culture and incubated for an additional 45 min at 37°C with shaking at 225 rpm. Arabinose induces protein synthesis and promotes abltide cell surface expression, whereas EDTA facilitates the peptide display. After induction, cells were kept at 4°C and used within 30 h. The same procedure was followed with *E. coli* expressing the eCPX scaffold, which was used as a control in the kinase assay.

### Kinase activity, incubation time, ATP and kinase concentration optimization

Thirty five µL of induced cells were used in each kinase reaction. Before starting the reaction, cells were spun down at 3000 g for 5 min at 4°C and washed with cold phosphate buffer solution (PBS; 137 mM NaCl, 2.7 mM KCl, 10 mM Na_2_HPO_4_, 1.8 mM KH_2_PO_4_, pH 7.4). Cells were pelleted, the supernatant was removed and stock solutions were added to have a final kinase reaction volume of 35 µL in all the conditions tested. All the reagents in the kinase reaction were solubilized in kinase buffer (NEBuffer) and the kinase reaction was started by addition of ATP from stock solutions with 10-fold concentration. The conditions and final concentrations of each reagent are as detailed below.

To evaluate the time-course for the phosphorylation of abltide by Abl TK, cells expressing abltide were resuspended with Abl TK 0.5 U/mL, and the kinase was activated by adding 500 µM ATP and incubated at 37°C for different times (0, 10, 20, 30, 40, 50, 60, 90 and 120 min) with gentle shaking. The effect of ATP concentration on kinase activity was tested by incubating the induced *E. coli* cells with 0.5 U/mL Abl TK at 37°C for 30 min with final ATP concentrations ranging from 500 µM to 0.5 µM. To choose the best kinase concentration, *E. coli* cells were incubated with kinase concentrations ranging from 2 U/mL to 0.015 U/mL and incubated for 30 min with 500 µM ATP. To stop the reaction, cells were immediately centrifuged at 4°C and washed with cold PBS.

After the reaction was stopped, phosphorylated abltide was detected by incubating the cells with 40 µL of biotinylated antibody anti-phosphotyrosine 4G10 (Merck Millipore, 1 µg/mL in PBS) at 4°C for 45 min with gentle shaking. Cells were pelleted by centrifugation and incubated for further 45 min at 4°C with 40 µL streptavidin-R-phycoerythrin (SAPE; Invitrogen, 5 µg/mL in PBS). Cells were centrifuged and resuspended with 500 µL of ice-cold PBS (∼10^7^ cells/mL) and analyzed by flow cytometry (FC). Each experiment was done in duplicate and repeated three times. Negative controls without kinase were also included to evaluate unspecific binding to the antibody or to SAPE. In addition, eCPX- displaying *E. coli* cells were treated in the same way to evaluate unspecific phosphorylation by kinase.

### Inhibition and competition assay

Inhibition of Abl TK activity was evaluated by incubating induced cells with several concentrations of imatinib (ranging from 32 to 0.008 µM). Competition with soluble abltide was evaluated with peptide concentrations ranging from 64 to 0.25 µM. The abltide analogue [Y4F,F8Y] was used as a control. Inhibitor, or peptide, were solubilized in kinase buffer and co-incubated with abltide-expressing cells, 0.5 U/mL kinase and 500 µM, or 50 µM, ATP for 30 min. The reaction was started with the addition of ATP and incubation at 37°C and stopped with centrifugation as described above. Samples were fluorescently-labelled and treated as described above. Each experiment was done in duplicate and repeated three times.

### Quantification by flow cytometry

FC measurements were performed with a BD FACSCanto II flow cytometer instrument (BD Biosciences). The population of cells was selected based on forward scatter and side scatter measurements. Cells with phosphorylated abltide were identified with fluorescence emission signal at 585/42 nm (excitation with 488 nm laser and detection at 585 nm with 42 nm bandpass); to gate out dark events and select the fluorescent cells, the background fluorescence emission of control cells (eCPX-displaying cells) was screened and a gate drawn to include only 0.2% or less of fluorescent cells. The mean fluorescence of the cell population and the percentage of fluorescent cells was determined by screening 100,000 cells per sample.

### Flow cytometry data analysis

The background fluorescence from unspecific binding was very low and was neglected. The mean fluorescence was normalized to the maximum response in each assay. The K_D_ (kinase or ATP concentration required to phosphorylate half of the abltide molecules in the sample) was calculated by fitting the plot of the normalized mean fluorescence versus Abl TK or ATP concentration to the sigmoidal dose-response with variable slope 
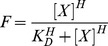
 in which F is the mean fluorescence, [X] is kinase or ATP concentration and H is the Hill slope.

The half-time (t_½_, time required to phosphorylate half of the abltide molecules displayed on the bacteria surface) was calculated assuming pseudo-first order association kinetics. The plot of the normalized mean fluorescence versus incubation time was fitted to the equation 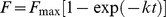, in which k is the rate constant and t is the time. The t_½_ was calculated from: 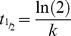


To calculate the percentage of inhibition, the mean fluorescence response obtained without inhibitor was considered as 100% of abltide phosphorylation and 0% of inhibition. The inhibitor concentration required to inhibit the phosphorylation of half of the abltide molecules (IC_50_) was calculated by fitting the inhibition percentage fluorescence as a function of inhibitor concentration to the sigmoidal dose-response with variable slope 
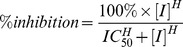
 in which [I] is the inhibitor concentration and H is the Hill slope.

## Results and Discussion

The *BacKin* assay is a novel kinase assay based on substrates displayed on a bacterial surface and FC detection. As a proof-of-concept, abltide, the optimal substrate of Abl kinase, was displayed on the surface of *E. coli* by fusing the C-terminus to the N-terminus of the eCPX display scaffold (see Figure 2A). After incubation with Abl kinase, phosphorylated abltide was fluorescently-labeled with biotinylated anti-phosphotyrosine and SAPE as summarized in Figure 2B. The extension of abltide phosphorylation correlated with the mean fluorescence of the sample, as quantified by FC.

### Validation of *BacKin* assay

**Figure 3:**
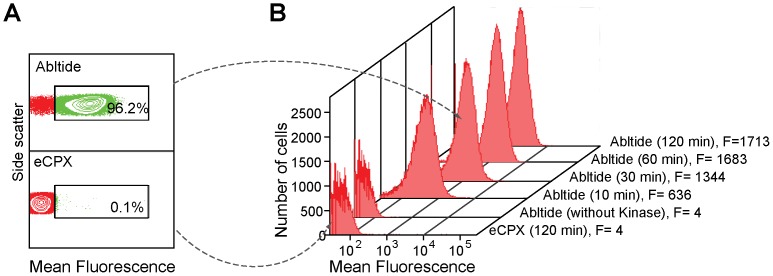
Validation of *BacKin* assay examined with flow cytometry. (A) Dotplot of side scatter and fluorescence emission intensity at 585/42 nm for eCPX-displaying cells and abltide-displaying cells. After treatment, the percentage of fluorescent cells (shown in green) is ∼0.1% in eCPX-displaying cells and > 96% in abltide-displaying cells. (B) Representative fluorescence intensity histograms for eCPX-displaying and abltide-displaying cells. The incubation time with kinase is given in brackets. All samples were fluorescently-labeled by incubation with biotin-anti-PY and SAPE. F is the mean fluorescence signal obtained for 100,000 cells analyzed.

To validate the *BacKin* assay, the fluorescence emission of abltide-expressing cells was compared with that for cells expressing eCPX scaffold (Figure 3). After 30 min incubation with Abl kinase, ∼96% of abltide-displaying cells were fluorescent, whereas eCPX-displaying cells were not fluorescent even after 2 h of incubation with kinase (Figure 3A). Furthermore, kinase-treated abltide-expressing cells had a large increase in mean fluorescence signal compared with eCPX-expressing cells, even with reaction times as short as 10 min (Figure 3B). Comparison of the fluorescence histograms obtained at different incubation times, revealed that with longer incubation there is an increase in the mean fluorescence, with the cell population showing a more homogeneous fluorescence signal.

The large difference in the fluorescence signal obtained with abltide- and eCPX-expressing cells confirms the high sensitivity to detect phosphorylated abltide with low unspecific phosphorylation of cells that express the eCPX scaffold. Unspecific binding of antibody or SAPE does not occur, as confirmed with abltide-expressing cells untreated with kinase (see Figure 3B).

The observed fluorescence profile reveals that: i) abltide is efficiently displayed at the cell surface; ii) surface exposed abltide can be efficiently phosphorylated by Abl kinase and iii) phosphorylated abltide can be fluorescently-labeled by anti-phosphotyrosine and SAPE.

### Characterization of Abl kinase activity using *BacKin* assay

**Figure 4:**
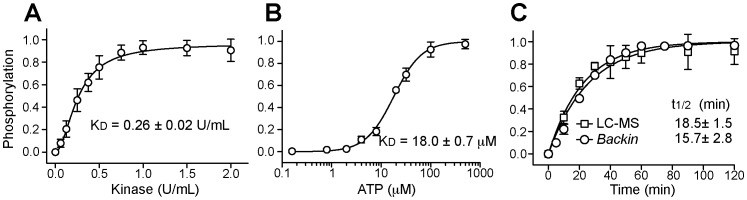
Characterization of Abl kinase using the *BacKin* assay. Mean fluorescence was converted into fraction of phosphorylation, assuming maximum response as 100% of abltide phosphorylation. Each data point is the average of three independent experiments±SD (A) Abltide phosphorylation catalyzed by increasing concentrations of Abl kinase in the presence of 500 µM ATP and incubated for 30 min at 37°C. The kinase concentration required to achieve half of the maximum response, K_D_±SD, was calculated by fitting a nonlinear sigmoidal curve. (B) ATP concentration effect on abltide phosphorylation catalyzed by 0.5 U/mL Abl Kinase for 30 min at 37°C. The ATP concentration required to achieve half of the maximum response, K_D_±SD, was calculated by fitting a nonlinear sigmoidal curve. (C) Time-course of abltide phosphorylation catalyzed by 0.5 U/mL Abl kinase in the presence of 500 µM ATP at 37°C obtained with *BacKin* assay (circles) or with LC-MS assay (squares). The time required to phosphorylate half of the abltide molecules, t_1/2_±SD, was calculated by fitting a pseudo-first order association kinetics.

To evaluate whether the *BacKin* assay could be used to characterize Abl TK activity, phosphorylation of abltide was studied as a function of kinase and ATP concentration and reaction time. The results in Figure 4A show an increase in abltide phosphorylation with the kinase concentration, followed by a steady state. The concentration required to phosphorylate half of the abltide molecules displayed on the surface of *E. coli* cells was found to be 0.26±0.02 U/mL. At a concentration equal to, or higher than 0.5 U/mL Abl TK, almost all the abltide molecules are phosphorylated; therefore, 0.5 U/mL Abl TK was the kinase concentration chosen to conduct the following kinase studies.

To examine the effect of ATP on the kinase reaction, concentrations ranging from 0.5 to 500 µM of ATP were tested in a 30 min reaction (Figure 4B). As expected, the phosphorylation of abltide catalyzed by Abl TK is ATP-dependent and no reaction occurs in the absence of ATP. The ATP concentration required to phosphorylate half of the abltide molecules was found to be 18.0±0.7 µM. To guarantee that ATP is not a limiting reagent 500 µM ATP was chosen to use in the following studies.

The kinetic of the reaction was followed for two hours and fitted with a pseudo-first order kinetics (Figure 4C). The rate constant of abltide phosphorylation catalyzed by 0.5 U/mL Abl TK was found to be (4.4±0.3)×10^−2^ min^−1^ and the half-time of the reaction was 15.7±2.8 min. Altogether the results in Figure 4 confirm that the *BacKin* assay can be used to characterize and evaluate Abl kinase activity under various conditions.

The reaction time-course was also followed using a cell-free assay and quantified by LC-MS. The reaction was conducted with 60 µM of soluble abltide, 500 µM ATP and 0.5 U/mL Abl TK (see Figure 4C). These conditions yielded a reaction rate of (3.8±0.1)×10^−2^ min^−1^) and a half-time of 18.5±1.5 min. The kinetic curves obtained with the *BacKin* assay and the LC-MS assay are identical, suggesting that under the conditions tested for the *BacKin* and cell-free assays the concentration of abltide is high enough to induce the maximum reaction velocity of Abl TK. These results confirm that Abl kinase activity can be efficiently measured using *BacKin* assay and results can be compared with other methods.

### Inhibition of Abl tyrosine kinase and substrate competition followed with *BacKin* assay

**Figure 5:**
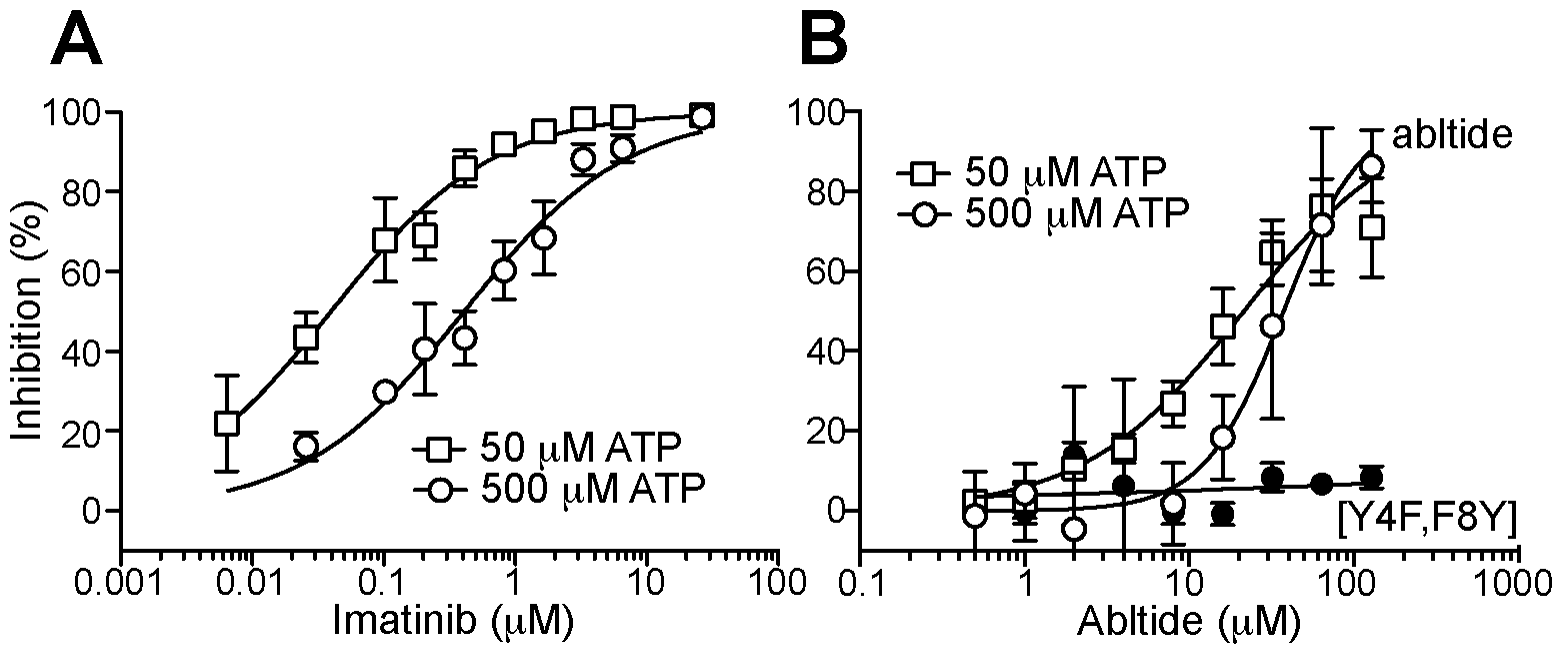
Kinase inhibition study using the *BacKin* assay. Inhibition of surface-displayed abltide phosphorylation by (A) imatinib or (B) soluble abltide. Reaction was catalyzed with 0.5 U/mL Abl kinase in the presence of 50 (squares) or 500 µM ATP (circles) and incubated for 30 min at 37°C. Mean fluorescence was converted into percentage of inhibition. Each data point represent the average of three independent experiments±SD and data were fit with a sigmoidal curve. The abltide analogue [Y4F,F8Y] is shown in black circles and was tested with 500 µM ATP, average of two replicates are shown.

To evaluate whether the *BacKin* assay can be used to characterize kinase inhibitors quantitatively, the kinase inhibition induced by the clinically used drug imatinib (Figure 5A) was studied. Imatinib dose-response was studied with 0.5 U/mL Abl TK and 500 µM, or 50 µM, ATP for 30 min. Abltide phosphorylation decreased with increasing concentrations of imatinib. Comparison of inhibition curves obtained with 50 µM vs. 500 µM ATP confirmed that inhibition of Abl kinase by imatinib is ATP-dependent with a 10-fold difference in IC_50_, but with identical Hill slope (Table 1). These results are consistent with imatinib and ATP competing for the same binding site and IC_50_ values obtained are comparable with previously reported values [20], [27], [28].

**Table 1:** Kinase inhibition parameters.[a]

		IC_50_ (μM)	H
Imatinib	50 µM ATP	0.040±0.004	0.71±0.05
	500 µM ATP	0.409±0.040	0.71±0.05
Abltide	50 µM ATP	21.51±2.64	0.89±0.10
	500 µM ATP	37.47±3.95	1.80±0.31

[a] Inhibitory concentration (IC_50_) and Hill slope (H) determined by fitting dose-response plots with a sigmoidal curve (see Figure 5).

The *BacKin* assay can also be used in substrate competition studies as shown here with soluble abltide and with the analogue [Y4F,F8Y] as a negative control (Figure 5B). Incubation of abltide-expressing cells with increasing concentrations of soluble abltide induced a decrease in the fluorescent signal, suggesting that the soluble peptide and the cell-immobilized peptide compete for the enzymatic catalytic center. The analogue [Y4F,F8Y], although having a very similar sequence to abltide and with a Tyr residue, is not phosphorylated by Abl kinase and did not inhibit kinase activity in the concentration range tested.

As expected, the effect of ATP concentration on the inhibition of Abl kinase by the substrate abltide is distinct from the effect induced by the ATP-competitor imatinib (see Figure 5 and Table 1). Whereas the dose-response curve for imatinib has the same Hill slope but a 10-fold difference in IC_50_, the inhibition curve profile induced by abltide differs approximately 2-fold in the Hill slope and IC_50_ values when 500 µM ATP is compared with 50 µM ATP. The ATP concentration affects enzymatic kinetics (see Figure 4B), which explains the differences in the abltide IC_50_ and Hill slope with ATP concentration.

### Considerations for optimization of *BacKin* assay

However good was the reproducibility obtained with the *BacKin* assays, pitfalls might arise from using live organisms to display the peptide substrate. In particular, variable density of expressed peptide on the bacterial membrane might occur. As the kinetics of enzymatic reactions are dependent on the substrate concentration, the amount of abltide molecules expressed on the bacteria influences the reaction. Nevertheless, a good reproducibility was obtained when growth and induction conditions were kept constant. Indeed larger variability was found when different batches of kinase or if different kinase buffers were used (e.g. commercially available vs. prepared in house).

Although in the current study the assay was conducted using a tube-based approach, in principle it can be optimised into an automated HTS using a 96-well plate configuration compatible with many FC instruments. With such a configuration the volume and kinase concentration per sample could be further decreased.

## Conclusion

Using Bcr-Abl TK, a recognized oncogenic target, we developed a new cost-effective kinase assay. This non-radioactive *BacKin* assay, based on detection of phosphorylation of peptide substrates displayed on the bacteria surface and quantification by FC, can be used to: i) characterize and measure kinase activity; ii) screen small-molecule or peptide-based inhibitors or substrates; iii) compare and identify kinase substrates and iv) obtain mechanistic information on inhibition modes of action.

Importantly, peptide libraries displayed on the surface of bacteria [29] can be used in this approach to identify kinase peptide substrate/inhibitors. Bacterial peptide libraries are readily amplified by growth and are self-renewing; therefore, *BacKin* assay can be used to screen such libraries in a cost effective way to identify potential peptide-based leads. Clones that express positive hits can be selected and isolated, individually tested and sequenced and developed as potential drug leads for cancer treatment. This is a major advantage compared with a LC-MS approach, which even though applicable to screen and identify peptide substrates, would require all peptides to be screened and sequenced individually and therefore would not be appropriate to screen large libraries.

Although the *BacKin* assay was developed with abltide-displaying cells and Abl kinase, it could be similarly applied to other kinases and their respective substrates. The development of efficient screening techniques for measuring kinase activity is of major importance for drug research, as malfunction of protein TK activity is a hallmark of numerous diseases, including cancers, diabetes and immune diseases.
